# Obstetrical Complications in Women with Endometriosis: A Cohort Study in Japan

**DOI:** 10.1371/journal.pone.0168476

**Published:** 2016-12-22

**Authors:** Takashi Harada, Fuminori Taniguchi, Kazunari Onishi, Youichi Kurozawa, Kunihiko Hayashi, Tasuku Harada, Japan Environment & Children’s Study Group

**Affiliations:** 1 Department of Obstetrics and Gynecology, Tottori University Faculty of Medicine, Yonago, Japan; 2 Department of Public Health, Tottori University Faculty of Medicine, Yonago, Japan; 3 Department of Laboratory Science and Environmental Health Sciences, Graduate School of Health Sciences, Gunma University, Maebashi, Japan; National Institute for Research in Reproductive Health, INDIA

## Abstract

**Background:**

Endometriosis, which occurs in approximately 10% of women of reproductive age, is defined as the presence of endometrial tissue outside the uterus. Women with endometriosis are more likely to have difficulty conceiving and tend to receive infertility treatment, including assisted reproductive technology (ART) therapy. There has not yet been a prospective cohort study examining the effects of endometriosis on pregnancy outcome in pregnant Japanese women.

**Methodology:**

This was a prospective cohort study of the incidence of obstetrical complications in women with endometriosis using data of the Japan Environment & Children’s Study (JECS). Included in this study were 9,186 pregnant women in the JECS with or without a history of endometriosis who gave birth or stillbirth or whose pregnancy was terminated with abortion between February and December 2011.

**Main Outcome Measures:**

The effects of endometriosis on pregnancy outcome.

**Results:**

Of the 9,186 pregnant women in the JECS, 4,119 (44.8%) had obstetrical complications; 330 participants reported a diagnosis of endometriosis before pregnancy, and these women were at higher risk for complications of pregnancy than those without a history of endometriosis (odds ratio (OR) = 1.50; 95% confidence interval (CI) 1.20 to 1.87). Logistic regression analyses showed that the adjusted OR for obstetrical complications of pregnant women who conceived naturally and had a history of endometriosis was 1.45 (CI 1.11 to 1.90). Among pregnant women with endometriosis, the ORs of preterm premature rupture of the membranes (PROM) and placenta previa were significantly higher compared with women never diagnosed with endometriosis who conceived naturally or conceived after infertility treatment, except for ART therapy (OR 2.14, CI 1.03–4.45 and OR 3.37, CI 1.32–8.65).

**Conclusions:**

This study showed that endometriosis significantly increased the incidence of preterm PROM and placenta previa after adjusting for confounding of the data by ART therapy.

## Introduction

Endometriosis is defined as the presence of endometrium-like tissue outside the uterus. The disease is common, affecting 10% of reproductive age women and 40% of women seeking infertility evaluation. Until recently, obstetricians and gynecologists had been unaware of the potential risks during pregnancy of patients with endometriosis. However, recent epidemiological studies reported an association between endometriosis and adverse pregnancy outcomes [1, 2]. Some studies reported increased incidences of preterm birth, pregnancy-induced hypertension (PIH), and small for gestational age (SGA) babies in women with endometriosis, suggesting that endometriosis affects pregnancy outcomes [1, 2].

Women with endometriosis are more likely to have difficulty conceiving and tend to receive infertility treatment, including assisted reproductive technology (ART) therapy, which in itself is a risk factor for preterm birth, PIH, and SGA babies [3, 4]. No prospective cohort study has yet examined the effects of endometriosis on pregnancy outcomes in pregnant Japanese women. Furthermore, it is unclear whether pregnancy outcomes in women with endometriosis are affected by ART.

The aim of this study was to determine the incidence of adverse pregnancy outcomes and the influence of endometriosis. The effects of endometriosis on pregnancy outcomes were examined by comparing women with or without endometriosis, using a cohort of 9,186 births from the first part of the data from the Japan Environment & Children’s Study (JECS).

## Subjects and Methods

### Data Sources

The purpose of the JECS, an ongoing prospective birth cohort study that began in 2011, is to evaluate the impact of various environmental factors on children’s health and development [5, 6]. A total of 100,000 children and their parents took part across 15 regions in Japan with follow-up programs to examine health periodically from the early stages of pregnancy until the participating child reaches 13 years of age. The present study is based on the dataset of jecs-ag-ai-20131008, which was released in October 2013. Enrollment started on 24 January 2011 and ended on 31 March 2014. All participants provided their written, informed consent. The current study is considered as a part of the JECS study. All adjunct studies are not required to have patient’s approval because it has been already written in the original consent.

The JECS protocol was approved by the Institutional Review Board (IRB) on epidemiological studies of the Ministry of the Environment (MOE) and the Ethics Committees of all participating institutions. The jecs-ag-ai-20131008 dataset does not contain any patient identifying information. This study was conducted under the approval of the JECS as an adjunct study. It has obtained the written consent approved by MOE, ensuring that they do not interfere with the main study of the JECS. We sought an approval from the ethics committee of the national center for japan environmental and children’s study.

The JECS is a national project which was designed to improve children’s health and development. The MOE instituted a general rule to open the jecs-ag-ai-20131008 dataset. Any researcher can use the dataset after seeking permission from the MOE. The email address is hoken-risuku@env.go.jp to contact details for inquiries about JECS.

The JECS participants were enrolled before delivery. A research coordinator described the JECS to the pregnant women after calculating the estimated date of delivery by ultrasound based on the crown lump length. In this study, each woman completed a questionnaire regarding her past history of endometriosis, reporting whether she had been diagnosed with endometriosis during the past year, ever had endometriosis, and ever received infertility treatment. This study did not take into account the period between diagnosis of endometriosis and the occurrence of pregnancy. Trained research coordinators collected data concerning obstetrical complications and neonatal outcomes from medical records in the obstetrics institutions.

### Participants

Included in this study were 9,186 pregnant women in the JECS with or without a history of endometriosis who gave birth, stillbirth, or whose pregnancy was terminated with abortion between February and December 2011. They were diagnosed with a single pregnancy in the first trimester by transvaginal ultrasound in the hospitals. Cases of multiple pregnancies were excluded.

### Clinical classification of participants

Maternal age was defined as age in completed years at the time of delivery and was categorized as <20, 20–24, 25–29, 30–34, 35–39, or 40 years and older. Women were classified as non-smokers, ex-smokers, current smokers, and those exposed to passive smoke, and they were sorted into < 3 days/week and ≥ 3 days/week. Alcohol drinking was defined as non-drinking, ex-drinking, and current drinking. ART therapy included information about in vitro fertilization (IVF) treatment, intracytoplasmic sperm injection (ICSI), frozen-thawed embryo transfer, and blastocyst embryo transfer for the present pregnancy. ART did not include artificial insemination with the husband’s semen (AIH).

Complications of pregnancy were characterized as threatened abortion, threatened premature delivery, premature rupture of the membranes (PROM), gestational diabetes, preeclampsia, placenta previa, placental abruption, fetal growth restriction (FGR), and non-reassuring fetal status (NRFS). Neonatal outcomes were defined as livebirth, abortion, and stillbirth.

### Diagnostic criteria for obstetrical complications

Threatened abortion was diagnosed by clinical symptoms, such as genital bleeding, lower abdominal pain, a shortened cervix before 22 weeks’ gestation, and threatened premature delivery from 22 to 37 weeks’ gestation. Non-reassuring fetal status (NRFS) was defined by doctors as an abnormal fetal heartbeat monitored during labor, classified as an FHR pattern level of 3 to 5 (mild, moderate, and severe variant patterns) using a combination of three factors: the baseline variability, the baseline, and the presence of various decelerations [7]. Preeclampsia was defined as persistently raised blood pressure ≥140/90 mmHg, occurring after >20 weeks of pregnancy in an otherwise normotensive woman, including gestational hypertension (preeclampsia without proteinuria), and gestational proteinuria (preeclampsia with proteinuria of ≥300 mg protein in 24 h) [7]. Preeclampsia with severe features was defined as severe blood pressure elevation (systolic blood pressure ≥ 160 mmHg or diastolic ≥ 110 mm Hg) and severe proteinuria (≥ 2,000 mg protein in 24 h).

The pregnant women with gestational diabetes (GD) were screened by the following stepwise method in Japan. Firstly, the random blood glucose levels were measured at early stage of pregnancy (each hospital should determine its own cut-off value). A 50-g glucose challenge test was given to the pregnant women (cut-off value ≥140 mg/dL) or the random glucose levels were measured for the second time (cut-off value ≥100 mg/dL) between 24 and 28 weeks. The pregnant women with a positive screening test result were tested for 75-g oral glucose tolerance test (OGTT). Gestational diabetes is defined as the pregnant woman having fulfilled one or more threshold values of a 75-g OGTT. Threshold values for 75-g OGTT are fasting plasma glucose (FPG) ≥ 92 mg/dL, 1-h PG ≥ 180 mg/dL, and 2-h PG ≥ 153 mg/dL [7]. FGR was defined as below the −1.5 SD value of the mean estimated fetal weight (EFW). Abortion was defined as termination of a pregnancy caused by spontaneous abortion or medical means prior to 22 weeks of gestation. Stillbirth is commonly defined as the death of a fetus at 22 or more weeks’ gestation that died before or during delivery.

### Statistical Analysis

The Wilcoxon rank-sum test and the chi-squared test were used to evaluate whether there were significant differences in age, smoking, passive smoking, alcohol drinking, gestational age, and other clinical characteristics between women with and without endometriosis. To compare the incidence of complications of pregnancy in women with and without endometriosis, Fisher’s exact test and logistic regression analysis were used. To examine the interaction between endometriosis and fertility treatment, all women were grouped into one of four combination groups: Group-A1, as the reference group, women without a history of endometriosis and without infertility treatment group; Group-A2, women with endometriosis and without infertility treatment; Group-A3, women without endometriosis and with infertility treatment; and Group-A4, women with endometriosis and with infertility treatment. Unconditional logistic regression models were used to estimate age-adjusted odds ratios (ORs) and their 95% confidence intervals (CIs). To examine the interaction between endometriosis and ART therapy, women were also grouped into four combination groups: Group-B1, as the reference group, women without a history of endometriosis who conceived naturally or conceived after infertility treatment, except for ART therapy; Group-B2, women with endometriosis and without ART therapy; Group-B3, women without endometriosis and with ART therapy; and Group-B4, women with both endometriosis and ART therapy. We restricted the analyses population to the pregnancies with complete covariate data. All analyses were performed using SAS V.9.4 (SAS Institute Inc., Cary, NC, USA). A value of p<0.05 was considered significant for every statistical analysis.

## Results

The participants’ characteristics, age, smoking, passive smoking, and alcohol drinking were similar between women with and without endometriosis (Table 1). A total of 9,186 pregnant women were enrolled whose pregnancy terminated between 1 February and 31 December 2011. Of the 9,186 participants, 330 reported a diagnosis of endometriosis before pregnancy; 266 conceived naturally (80.6%), 29 received ART therapy for infertility (8.8%), 13 pregnant women received intracytoplasmic sperm injection, and 16 patients underwent IVF. Twenty pregnant women answered that a blastocyst was transferred into the uterus at the time of ART.

**Table 1:** Clinical Characteristics of Women with and without Endometriosis.

Past history of endometriosis	Positive(n = 330)	Negative(n = 8,856)	P Value
**Maternal age (y)**			
< 20	1 (0.3)	76 (0.9)	<0.01^c^
20–24	9 (2.7)	683 (7.7)	
25–29	52 (15.8)	2,117 (23.9)	
30–34	110 (33.3)	2,725 (30.8)	
35–39	95 (28.8)	1,663 (18.8)	
≥ 40	15 (4.5)	305 (3.4)	
Missing	48	1,287	
**Smoking**			
Non-	183 (55.5)	4,989 (56.3)	0.78^a^
Ex-	130 (39.4)	3,302 (37.3)	
Current	17 (5.2)	492 (5.6)	
Missing	0	73	
**Passive smoking**			
<3 days/week	235 (71.2)	6,230 (70.3)	0.83^a^
≥3 days/week	95 (28.8)	2,585 (29.2)	
Missing	0	41	
**Alcohol drinking**			
Non-	100 (30.3)	2,927 (33.1)	0.53^a^
Ex-	208 (63.0)	5,314 (60.0)	
Current	12 (3.6)	345 (3.9)	
Missing	10	270	
**Gestational age**			
weeks, median [range]	39.0 [15.0–42.1]	39.3 [7.4–42.3]	<0.01^c^
< 22	5 (1.5)	78 (0.9)	
22–37	34 (10.3)	504 (5.7)	
37–42	289 (87.6)	8227 (92.9)	
≥ 42	1 (0.3)	17 (0.2)	
Missing	1	30	
**Parity**			
0	141 (42.7)	3,192 (36.0)	<0.01^c^
1	115 (34.8)	3,369 (38.0)	
2	41 (12.4)	1,435 (16.2)	
> 3	12 (3.6)	409 (4.6)	
Missing	21 (6.4)	451 (5.1)	
**Mode of delivery**			
Vaginal delivery	244 (73.9)	7,200 (81.3)	<0.01^a^
Cesarean section	85 (25.8)	1,570 (17.7)	
Missing	1	86	
**Infertility treatment**			
None	266 (80.6)	8,294 (93.7)	<0.01^a^
Ovulation induction	31	370	<0.01^b^
Artificial insemination	16	136	0.88^b^
ART	29 (8.8)	192 (2.2)	0.10^b^
ICSI	13	96	0.69^b^
Blastocyst transfer	20	122	0.68^b^
Other	12	95	0.73^b^
Missing	0	0	
**Neonate**			
Female sex	160 (48.5)	4,285 (48.4)	0.95^a^
Birth weight			
g, mean ± SD	2984 ± 588.2	3010 ± 504.4	0.09^c^

Data expressed as n (%).

a, Chi-squared test

b, Fisher's exact test

c, Wilcoxon rank-sum test

A total of 4,119 (44.8%) of the 9,186 JECS participants were diagnosed with obstetrical complications; 180 of 330 women with endometriosis had obstetrical complications (54.5%). Of 8,856 women without endometriosis, 3,939 (44.5%) had complications of pregnancy (Table 2).

**Table 2:** Types of Obstetrical Complications and Neonatal Outcomes.

Past history of endometriosis	Positive	Negative	P Value
	n (%)	n (%)	
**Obstetrical complications**			
Positive	180 (54.5)	3,939 (44.5)	<0.05^b^
Negative	150 (45.5)	4,917 (55.5)	
**Threatened abortion**			
Positive	41 (12.4)	921 (10.4)	0.23^b^
Negative	289 (87.6)	7,935 (89.6)	
**Threatened premature delivery**			
Positive	85 (25.8)	1,647 (18.6)	<0.05^b^
Negative	245 (74.2)	7,209 (81.4)	
**Premature rupture of the membranes**			
Positive			0.07^a^
Preterm PROM	10 (3.0)	124 (1.4)	
Term PROM	13 (3.9)	443 (5.0)	
Unknown	4 (1.2)	155 (1.8)	
Negative	303 (91.8)	8,134 (91.8)	
**Gestational diabetes**			
Positive	12 (3.6)	207 (2.3)	0.14^b^
Negative	318 (96.4)	8,649 (97.7)	
**Preeclampsia(mild)**			
Positive	4 (1.2)	190 (2.1)	0.33^b^
Negative	326 (98.8)	8,666 (97.9)	
**Preeclampsia(severe)**			
Positive	4 (1.2)	91 (1.0)	0.59^b^
Negative	326 (98.8)	8,765 (99.0)	
**Placenta previa**			
Positive	12 (3.6)	52 (0.6)	<0.05^b^
Negative	318 (96.4)	8,804 (99.4)	
**Abruption of the placenta**			
Positive	5 (1.5)	34 (0.4)	<0.05^b^
Negative	325 (98.5)	8,822 (99.6)	
**Fetal growth restriction**			
Positive	10 (3.0)	199 (2.2)	0.34^b^
Negative	320 (97.0)	8,657 (97.8)	
**Non-reassuring fetal status**			
Positive	7 (2.1)	141 (1.6)	0.38^b^
Negative	323 (97.9)	8,715 (98.4)	
**Obstetrical outcomes**			
Livebirth	324 (98.2)	8,737 (98.7)	0.46^b^
Stillbirth/Abortion	6 (1.8)	119 (1.3)	

a, Chi-squared test

b, Fisher's exact test

As shown in Table 3, women with endometriosis were at increased risk for complications of pregnancy when compared with those without endometriosis (OR = 1.50; 95% CI = 1.20–1.87). Interestingly preterm PROM, placenta previa and placental abruption appeared to increase in women with endometriosis (OR = 2.17; 95% CI = 1.13–4.17, OR = 6.39; 95% CI = 3.38–12.09 and OR = 3.99; 95% CI = 1.55–10.27). The incidence of preeclampsia did not increase in the endometriosis group.

**Table 3:** Relative Risk of Obstetrical Complications and Neonatal Outcomes.

	Endometriosis	Endometriosis multivariable-adjusted^a^
	Crude OR (95% CI)	Adjusted OR (95% CI)
Obstetrical complications	1.50 (1.20–1.87)^b^	1.50 (1.18–1.92)^b^
Threatened abortion	1.22 (0.88–1.71)	1.28 (0.88–1.85)
Threatened premature delivery	1.52 (1.18–1.96)^b^	1.53 (1.16–2.03)^b^
Preterm PROM	2.17 (1.13–4.17)^b^	1.84 (0.84–4.01)
Gestational diabetes	1.58 (0.87–2.85)	1.35 (0.70–2.59)
Preeclampsia(mild)	0.56 (0.21–1.52)	0.47 (0.15–1.48)
Preeclampsia(severe)	1.18 (0.43–3.24)	1.25 (0.45–3.45)
Placenta previa	6.39 (3.38–12.09)^b^	6.42 (3.25–12.65)^b^
Placental abruption	3.99 (1.55–10.27)^b^	3.45 (1.19–10.01)^b^
Fetal growth restriction	1.36 (0.71–2.59)	1.60 (0.83–3.06)
Non-reassuring fetal status	1.34 (0.62–2.89)	1.51 (0.65–3.48)

OR, odds ratio; CI, confidence interval; n/a, not applicable

a Multivariable-adjusted by age, smoking (Non-,Ex-,Current), passive smoking (< 3 days/week, ≥3 days/week), alcohol drinking (Non-,Ex-, Current)

b P < .05

On multivariable analysis, adjustment for confounders known to be associated with adverse pregnancy outcomes, such as maternal age, smoking habits, and alcohol drinking, was performed. In the adjusted model, women with endometriosis were at increased risk of threatened preterm delivery (OR = 1.53; 95% CI, 1.16–2.03), placenta previa (OR = 6.42; 95% CI = 3.25–12.65), and placental abruption (OR = 3.45; 95% CI = 1.19–10.01). However, endometriosis did not change the risk for threatened abortion and preterm PROM. Furthermore, no significant difference was found in the incidence of fetal growth restriction and NRFS.

To remove the influence of infertility treatment, logistic regression analyses were performed for the four combination groups by endometriosis and infertility treatment; the adjusted OR for obstetrical complications in pregnant women with endometriosis who conceived naturally (Group-A2) was 1.45 (95% CI = 1.11–1.90). Moreover, the adjusted ORs for preterm PROM, placenta previa, and placental abruption associated with endometriosis among women without infertility treatment were 2.51 (95% CI = 1.20–5.23), 3.31 (95% CI = 1.16–9.41), and 3.43 (95% CI = 1.03–11.48) (S1 Table). Among pregnant women with endometriosis, the ORs of preterm PROM and placenta previa were significantly higher compared with women never diagnosed with endometriosis who conceived naturally or conceived after infertility treatment, except for ART therapy (S1 Table).

## Discussion

In this study, two important clinical observations from a large cohort study were substantiated: 1) women with endometriosis have an increased risk of obstetrical complications, such as preterm PROM and placenta previa; and 2) women with endometriosis, regardless of receiving ART therapy, have an increased risk of preterm PROM and placenta previa.

The present study is the first to show a significant impact of endometriosis on the incidence of obstetrical complications after adjusting for the confounding of ART. Although Maggiore et al reported that ovarian endometriosis do not impair spontaneous ovulation [8], it is not controversial that endometriosis is a cause of infertility and a large number of women who are infertile because of endometriosis conceived using ART.

Furthermore, singleton pregnancies conceived using ART are at higher risk of obstetrical complications than those conceived naturally [3]. However, several reports concluded that there is no difference in pregnancy outcomes in women with endometriosis [9–11]. Benaglia et al. reported that women with endometriomas achieving pregnancy through IVF did not seem to be exposed to a significantly increased risk of obstetrical complications [10]. Because they selected only 234 subjects conceiving through ART therapy, they did not take into account the risk of ART, which can lead to threatened premature delivery. Mekaru et al. selected 108 cases who were surgically diagnosed with endometriosis and had no ART therapy. The sample size was too small to evaluate the OR of placenta previa, which has a 0.5% prevalence, and other obstetrical complications [11]. In contrast, Stephansson et al. [2], Aris et al. [12], and the present study, which enrolled almost 10,000 patients, demonstrated that endometriosis significantly influences obstetrical complications.

Endometriosis is a complex disease with multiple pathophysiological mechanisms. The eutopic endometrium of women with endometriosis has been shown to be functionally abnormal, exhibiting subtle but biologically important molecular abnormalities, including an increased production of estrogen, cytokines, prostaglandins, and metalloproteinases [13, 14]. The increased expression of cyclooxygenase-2 (COX-2) causes augmented secretion of prostaglandin E2 (PGE2) and prostaglandin F2α (PGF2α) in the uterine and endometriotic tissues of women with endometriosis [15]. Aromatase, a local enzyme, is hyperexpressed in endometriosis, leading to abnormal biosynthesis of estradiol (E2), which in turn, increases PGE2 formation by stimulating COX-2 expression, resulting in a positive feed-forward loop between estrogens and PGs that favors the proliferative and inflammatory characteristics of endometriosis [16,17]. Many studies have demonstrated that the levels of PGs and cytokines in women with endometriosis were increased in peritoneal fluid [18–21]. Pro-inflammatory mediators, such as PGE2, COX-2, and interleukin-8, reportedly cause uterine muscle contractions and cervical ripening and are linked to preterm birth [22].

Brosens et al. reported that the adverse impact of pelvic endometriosis on uterine function before conception may also interfere with subsequent deep placentation, including preterm birth and antepartum hemorrhage. They considered the pathological pathway, including the altered junctional zone myometrium that causes the clinical consequences of uterine dysfunction associated with pelvic endometriosis [23]. Thus, both the coexistence of endometriosis and pregnancy and endometriosis before pregnancy may affect obstetrical complications.

The adjusted OR for placenta previa associated with endometriosis among women without infertility treatment was 3.31. A recent systematic review on endometriosis during pregnancy supported the idea that pregnant women with endometriosis had an increased risk of placenta previa [24]. Additionally, placenta previa was significantly increased with a previous history of endometriosis as compared with fertile and infertile women conceiving without ART therapy (OR = 3.37). The risk of placenta previa in pregnancies following ART is considerably higher than that in pregnancies following natural conception [25]. Many reports suggested that factors directly related to reproduction technology contribute to the increased risk. The stimulation protocol used in ART therapy frequently results in very high levels of E2 and progesterone that induce morphological and structural changes and disturbed expressions of relevant genes in the endometrium [26]. In ART therapy, embryos are placed in the uterine cavity by the transcervical route using a catheter. This procedure may induce uterine contraction, possibly due to the release of PGs after mechanical stimulation of the internal cervical os [27–29]. Conceivably, these mechanically induced uterine contractions could lead to higher frequencies of implantation in the lower uterine segment and thereby increase the risk of placenta previa. Chronic inflammation and the altered junctional zone may comprise the biochemical background for placenta previa in women with endometriosis. Furthermore, the rate of placenta previa in endometriosis patients without ART therapy was also elevated.

In the present study, the past history of endometriosis was determined by questionnaire, and a pregnant woman reported whether she had been diagnosed with endometriosis during her lifetime. Therefore, the diagnostic accuracy of endometriosis was reflected in a self-reported questionnaire. Endometriosis can be associated with a wide variety of symptoms, or it may be asymptomatic and incidentally observed at laparoscopy or exploratory surgery. Laparoscopic surgery and histological examination are strictly required for a precise diagnosis of endometriosis. In contrast, Japanese gynecologists routinely examine the patient’s uterus and ovaries in the first examination using transvaginal ultrasonography. The diagnostic accuracy of endometriosis, particularly in patients with ovarian endometrioma by transvaginal ultrasonography, is about 90% without surgery [30].

One of the weak points of this study is that the diagnostic accuracy of endometriosis is just referred by the self-reported questionnaire. Little information is available concerning with the medical records of the participants for endometriosis. It is unclear how many out of 330 women had active endometriosis during their pregnancies.

It is a widely-accepted fact that 40–50% of women with endometriosis suffer from infertility [31]. However, the JECS participants were recruited in early pregnancy at obstetric facilities, thereby 80.6% of women in the endometriosis group conceived naturally in this study. There is the possibility that we evaluated the obstetrical complications in subjects with 50 to 60% of fertile women affected with endometriosis.

In the present study, the data of obstetrical complications and neonatal outcomes were collected prospectively by trained research coordinators for all puerperal patients from the medical records. Therefore, we expect the self-reported questionnaire and the outcome of delivery to be accurate. This study did not take into account whether the affected women were treated for endometriosis before pregnancy and what kind of treatment was given. Furthermore, it is unclear whether obstetrical complications are affected by pre-pregnancy treatment or the coexistence of endometriosis during pregnancy.

## Conclusions

The present study demonstrated that preterm PROM and placenta previa are more frequent complications of pregnancy in women with a history of endometriosis. The present study is the first to show a significant impact of endometriosis, causing an increased incidence of preterm PROM and placenta previa after adjusting for the confounding of ART.

One of the weak points of this study is the diagnosis of endometriosis was based on self-reporting by the participants. The authors did not have an access to the medical records of the participants. It was not feasible to know how many women had active endometriosis during their pregnancies.

## Supporting Information

S1 Table Relative Risk of Obstetrical Complications Associated with Fertility Treatment. (PDF)
