# Supplementary material for: Obstetrical Complications in Women with Endometriosis: A Cohort Study in Japan
Source: PLoS One. 2016 Dec 22;11(12):e0168476. doi: 10.1371/journal.pone.0168476 (PMC5179019; doi:10.1371/journal.pone.0168476)
Supplement: S1 Table — (PDF) [file pone.0168476.s001.pdf]

S1 Table. Relative Risk of Obstetrical Complications Associated with Fertility Treatment

Group	A1[a]	A2	A3	A4	B1[b]	B2	B3	B4
Fertility treatment	negative	negative	positive	positive	ART negative	ART negative	ART	ART
Past history of endometriosis	negative	positive	negative	positive	negative	positive	negative	positive
		aOR (95% CI)	aOR (95% CI)	aOR (95% CI)		aOR (95% CI)	aOR (95% CI)	aOR (95% CI)
Obstetrical complications	reference	1.45 (1.11-1.90)[c]	1.13 (0.94-1.35)	1.93 (1.15-3.24)[c]	reference	1.40 (1.09-1.80)[c]	1.36 (1.01-1.84)[c]	4.40 (1.77-10.93)[c]
Threatened abortion	reference	1.14 (0.75-1.74)	1.16 (0.86-1.55)	1.80 (0.91-3.57)	reference	1.31 (0.89-1.91)	2.00 (1.34-2.99)[c]	1.18 (0.35-3.95)
Threatened premature delivery	reference	1.53 (1.13-2.08)[c]	0.97 (0.76-1.23)	1.61 (0.91-2.87)	reference	1.55 (1.16-2.07)[c]	1.10 (0.75-1.61)	1.62 (0.68-3.85)
Preterm PROM	reference	2.51 (1.2-5.23)[c]	1.25 (0.62-2.52)	2.25 (0.54-9.42)	reference	2.14 (1.03-4.45)[c]	1.19 (0.37-3.86)	5.20 (1.2-22.49)[c]
Gestational diabetes	reference	1.63 (0.82-3.24)	1.53 (0.96-2.44)	0.55 (0.07-3.97)	reference	1.34 (0.68-2.67)	1.46 (0.72-2.94)	1.13 (0.15-8.43)
Preeclampsia（mild)	reference	0.64 (0.2-2.04)	1.65 (0.99-2.74)	n/a	reference	0.54 (0.17-1.70)	1.61 (0.73-3.54)	n/a
Preeclampsia（severe)	reference	1.26 (0.39-4.05)	1.70 (0.86-3.36)	1.36 (0.19-10.02)	reference	1.06 (0.33-3.40)	2.10 (0.82-5.38)	2.93 (0.39-22.06)
Placenta previa	reference	3.31 (1.16-9.41)[c]	2.64 (1.2-5.83)[c]	20.82 (8.72-49.69)[c]	reference	3.37 (1.32-8.65)[c]	3.17 (1.08-9.27)[c]	41.59 (15.55-111.27)[c]
Placental abruption	reference	3.43 (1.03-11.48)[c]	0.91 (0.21-3.91)	3.72 (0.49-28.19)	reference	2.99 (0.9-9.97)	1.17 (0.15-8.93)	8.35 (1.07-65.05)c
Fetal growth restriction	reference	1.42 (0.66-3.06)	1.41 (0.82-2.43)	2.37 (0.73-7.70)	reference	1.52 (0.77-3.02)	0.50 (0.12-2.07)	1.70 (0.23-12.64)
Non-reassuring fetal status	reference	1.61 (0.65-4.00)	1.14 (0.55-2.39)	1.17 (0.16-8.57)	reference	1.67 (0.72-3.84)	1.20 (0.37-3.86)	n/a

Estimates are based on models that adjust for age. Note: aOR, adjusted odds ratio; CI, confidence interval; n/a, not applicable.

[a] Group-A1: women without a history of endometriosis and without infertility treatment

[b] Group-B1: women without a history of endometriosis who conceived naturally or conceived after infertility treatment except for ART therapy

[c] P value < .05
